# Laparoscopic ventral onlay ureteroplasty with buccal mucosa graft for complex proximal ureteral stricture

**DOI:** 10.1590/S1677-5538.IBJU.2023.0170

**Published:** 2023-07-10

**Authors:** B. G. Guliev, Boris Komyakov, Zhaloliddin Avazkhanov, Maksim Shevnin, Ali Talyshinskii

**Affiliations:** 1 North-Western State Medical University named after I. I. Mechnikov Department of Urology Saint Petersburg Russia Department of Urology, North-Western State Medical University named after I. I. Mechnikov, Saint Petersburg, Russia; 2 Urology Center with robot-assisted surgery of the Mariinsky Hospital Department of Urology Saint Petersburg Russia Department of Urology, Urology Center with robot-assisted surgery of the Mariinsky Hospital; Saint Petersburg, Russia

**Keywords:** Urethral Stricture, Mouth Mucosa, Laparoscopy

## Abstract

**Introduction:**

There is lack of papers dedicated to the laparoscopic buccal mucosa graft (BMG) ureteroplasty of the complex upper ureteral stricture. The aim of this study is to evaluate the results of laparoscopic BMG ureteroplasty in patients with complex proximal ureteral stricture.

**Material and methods:**

Twenty-four patients underwent laparoscopic ventral onlay BMG ureteroplasty for long or recurrent proximal ureteral stricture not amenable to uretero-ureteral anastomosis over 2019-2022. Patient demographics, operative time, estimated blood loss, length of stay, follow-up, intra- and postoperative complication rate and percentage of stricture-free at last visit were analyzed.

**Results:**

The mean stricture length was 3.6 cm. The mean operative time was 208.3 min, while mean blood loss was 75.8 mL. The length of hospital stay was 7.3 days. No intraoperative complications were observed. Postoperatively, seven patients developed complications (29.2%). Five patients experienced a Grade II (according to Clavien nomenclature). Two patients developed a Grade IIIa complication, which included leakage of the anastomosis site. The mean follow-up was on the 22 months with stricture free rate 87.5%.

**Conclusion:**

Patients with proximal ureteral strictures could be effectively treated by laparoscopic ventral onlay ureteroplasty with a buccal mucosa graft.

## INTRODUCTION

Long ureteral strictures represent a complicated dilemma that requires the significant experience and extensive surgical arsenal of urologists. The choice of surgical strategy depends on properties of the diseased ureter, mainly the localization and length of the stricture. Basically, the following procedures are proposed to be effective in the case of complex proximal ureteral stricture: ileal substitution of the ureter, ureterocalicostomy, downward nephropexy and auto-transplantation (1–3). However, associated drawbacks motivate urologists to seek new approaches to correcting such strictures, in particular with the help of grafts.

One of these is the buccal mucosa graft (BMG), which has been demonstrated over the past few decades to have excellent outcomes in patients with urethral strictures. The first BMG ureteroplasty in humans was described in 1999 (4). However, interest in this technique has renewed over the past decade, and the technique is now reported in numerous articles regarding its utility in open and robotic approaches (5–7). Unfortunately, there is a lack of papers dedicated to the laparoscopic BMG ureteroplasty of the complex upper ureteral stricture, being necessary to build a confident opinion on the pros and cons of different approaches (8, 9).

Therefore, we hypothesized that laparoscopic BMG ureteroplasty can be effectively and safely used for patients with complex proximal ureteral strictures, when ureteroureterostomy is impossible, or with recurrent ureteral strictures. Herein, we present the single-center experience in performing laparoscopic BMG ureteroplasty in 24 patients with a complex proximal ureteral stricture.

## MATERIAL AND METHODS

**Figure 1:**
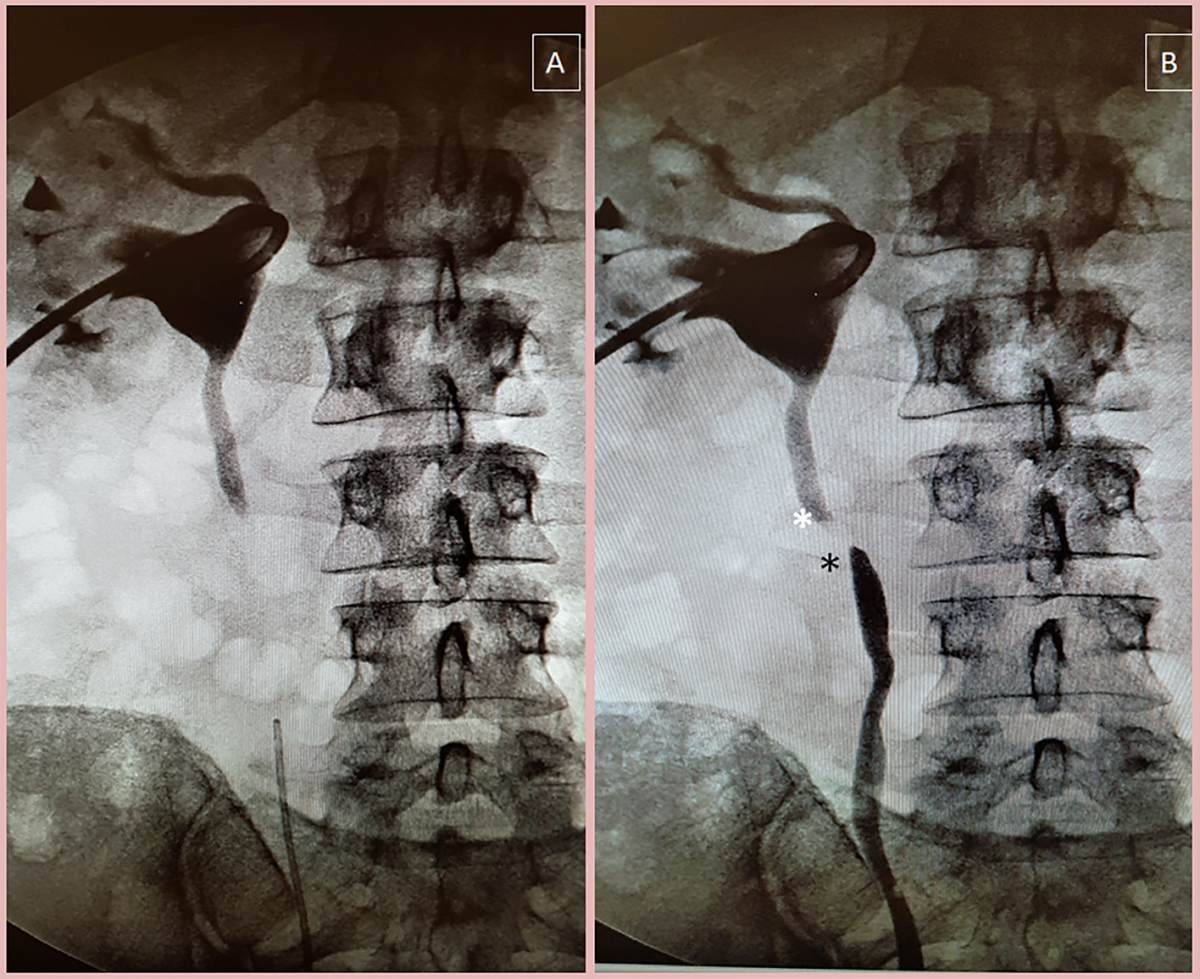
Preoperative definition of ureteral stricture length and location.

Retrospectively, data from 24 patients who underwent laparoscopic BMG ureteroplasty between 2019 and 2022 at a single institution were collected and analyzed after local ethical committee approval (PG071). All subjects provided written informed consent for inclusion in the study. All procedures performed in studies involving human participants were in accordance with the 1964 Helsinki Declaration and its later amendments or comparable ethical standards. The eligible criteria for this technique were a benign proximal ureteral stricture that was not amenable to primary anastomosis due to stricture length or extensive fibrosis. In all cases, the technique of choice was ventral onlay ureteroplasty, so patients were excluded if they had a complete absence of a large (>5 cm) portion of the ureter, such as after oncologic resection or ureteral avulsion. Those patients underwent alternative reconstructive techniques, such as a tapered bowel according to the Monti technique or tubular ileal segment substitution. Each patient underwent retrograde and antegrade (in the presence of a nephrostomy tube) pyelography to delineate stricture localization and length (Figure-1).

Patient demographics and preoperative characteristics are detailed in Table-1. Iatrogenic strictures were caused by lithotripsy and extraction of ureteral stones. The mean stricture length was 3.6±1.3 cm. Five patients had previously undergone ureteroureterostomies.

**Table 1:** Patient demographics and preoperative characteristics.

Parameter		n (%)
Number of patients		24 (100)
Proximal ureteral stricture		24 (100)
Age, years, mean±SD (range)		44.8±14.7 (19-74)
**Sex**		
	Male	16 (66.7)
	Female	8 (33.3)
BMI, kg/cm^2^, mean±SD (range)		26.7±2.8 (21.2–31.6)
Preoperative presence of nephrostomy tube		14 (58,3%)
**Diseased side**		
	Left	13 (54.2)
	Right	11 (45.8)
**Stricture etiology**		
	Iatrogenic	18 (75)
	Idiopathic	5 (20.8)
	Impacted stone	1 (4.2)
Previous failed ureteroplasty		5 (20.8)
Stricture length, cm, mean±SD (range)		3.6±1.3 (2.5-8)

## SURGICAL TECHNIQUE

Procedures were performed in the lateral decubitus position, while the legs were placed in a modified lithotomy position to allow for simultaneous ureteroscopy. The endotracheal tube was fixed on the dependent side of the mouth, and the mouth was draped separately from the abdominal field in preparation for BMG harvest, which may be completed after laparoscopic dissection of the ureter and after defining the diseased ureter length.

After sterile preparation and draping, a 12-mm incision was made about two fingerbreadths lateral to the umbilicus. The dermis and subcutaneous tissues areas were dissected with electrocautery, the rectus fascia was identified and lifted, and a Veress needle was used to establish a pneumoperitoneum of 10-15 mm Hg. The camera was then inserted, and the abdomen was inspected for access-related injuries or bowel adhesions. The triangulation rule was followed to place two additional accessory trocars at least four fingerbreadths from the primary trocar. A 12-mm trocar was placed cranially, and a 5-mm trocar was placed caudally and laterally at least four fingerbreadths away from the initial periumbilical trocar, also following the triangulation rule. Moreover, an additional 5-mm assistant trocar was placed four fingerbreadths caudally to the primary periumbilical trocar. The most cranial trocar was used for the camera while the other periumbilical and lower abdominal trocars were used to place working instruments.

**Figure 2:**
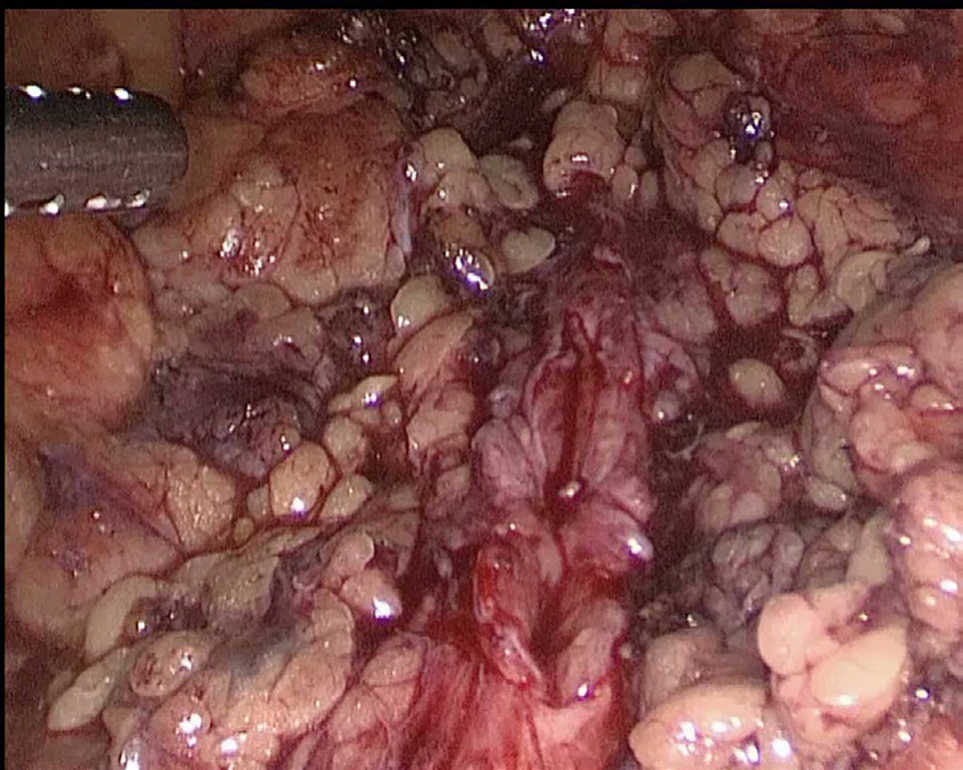
Opened ureter in the site of stricture. Expanded proximally and distally to healthy tissues.

Behind incision of the line of Toldt and medialization of the colon, Gerota fascia was exposed and opened. Intraoperative ureteroscopy was used to identify the stricture. The diseased ureter was dissected, paying attention to safe, healthy tissue as much as possible (Figure-2). The length of the stricture was determined using a ureteral catheter by placing it close to the stricture.

After the steps above, BMG harvesting was performed. First, the head was placed in the flank position, followed by cheek elevation, and the Stenson's duct was identified. 1% lidocaine and epinephrine were used for hydrodissection of the buccal mucosa. The graft size was tailored to the length of the ureteral defect. The defect in the cheek mucosa was closed with a continuous suture.

**Figure 3:**
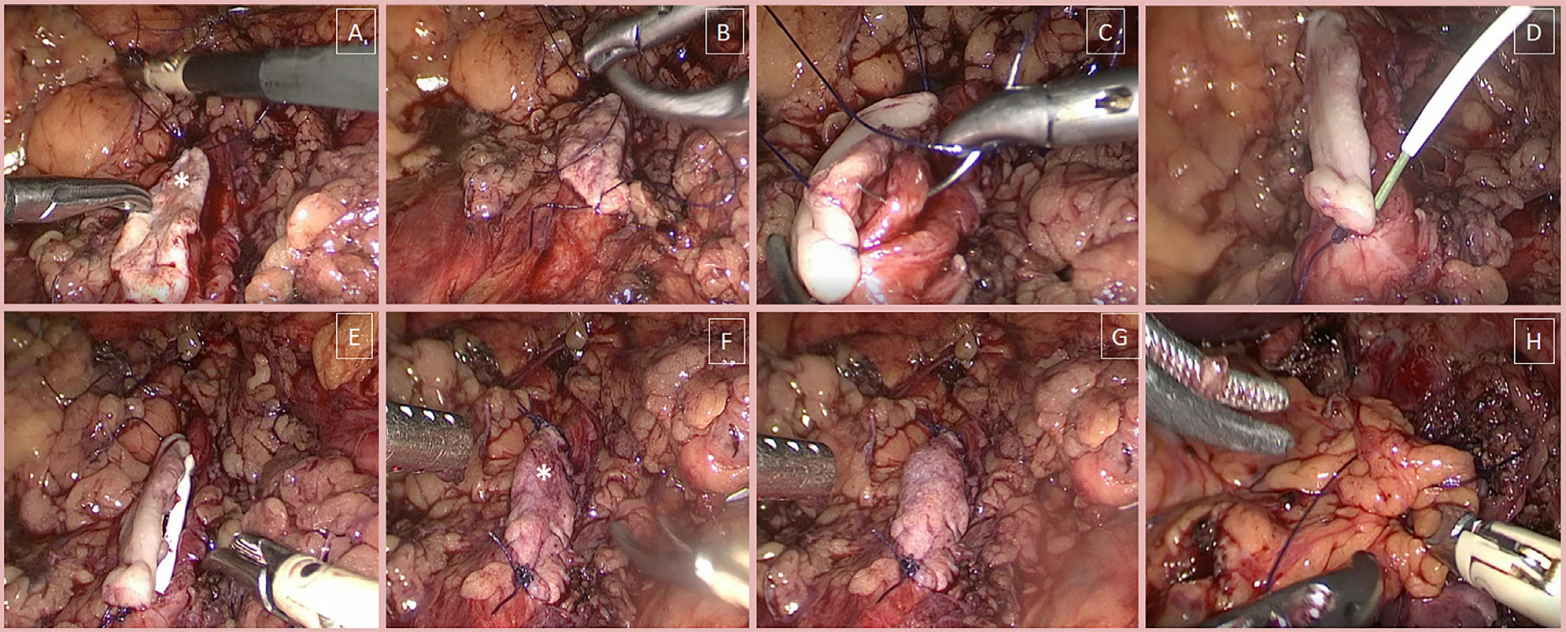
Step-by-step technique to perform ventral onlay BMG ureteroplasty.

Subsequently, the submucosal tissue was cleaned off the BMG, and the graft was passed through the trocar into the abdominal cavity. Vicryl 4/0 was used to sew apical angle of the graft to the superior aspect of the dissected ureter (Figure-3A). The graft was sutured distally to the ureteral defect (Figure-3B). A continuous suture was created between the buccal flap and the lateral margin of the ureter down to lower angle (Figure-3C).

Then, a 6Fr ureteral stent was introduced over the guidewire in distal ureter followed by guidewire removal (Figure-3D). The proximal aspect of the stent was inserted into the renal cavity (Figure-3E). Another vicryl 4/0 was used to suture the medial border of the dissected ureter with a buccal flap (Figure-3F). The watertightness of the anastomosis was checked by filling the collecting system with sterile fluid through the ureteroscope (Figure-3G). Then, the ureteroplasty field was wrapped in omentum or perirenal fat to improve the vascularity of the graft (Figure-3H). The wrap was fixed with knotty sutures to the surgical zone. A drain was placed adjacent to the anastomosis. After surgery, the nephrostomy tube was not closed to keep the pressure in the upper urinary tract low.

In the early postoperative period, patients were monitored for blood tests and ultrasound. Patients received anti-inflammatory and antibacterial therapy. The ureteral stent was removed after 6-8 weeks. Patients with a nephrostomy tube underwent antegrade pyelography. Then, the nephrostomy tube was removed. It should be noted that all procedures were performed by the same surgeon (G.B.) with experience in performing laparoscopic ureteroplasty in >100 cases (including ileal substitution, ureterocalicostomy, etc.).

Patient demographics, intra- and postoperative characteristics, follow-up duration, and percentage of stricture-free at the last visit, as well as complication rate according to Clavien-Dindo nomenclature, were calculated. The grade of hydronephrosis was assessed according to the SFU classification. A good postoperative outcome was considered to be the absence of any symptoms, the absence of hydronephrosis or grade 1, and the absence of nephrostomy drainage, provided that the contrast agent freely passes into the bladder through the ureter and the surgical site.

## Statistical Analysis

SPSS statistical software version 26.0 (IBM, Chicago, USA) was used for statistical analysis. Continuous data were presented as a mean and standard deviation according to data distribution, which was assessed via the Kolmogorov-Smirnov test. A range of values was also presented. Nominal data were presented as number and percentage. Depending on the type of data, we used the paired-samples t-test or McNemar's test for statistical analysis. Nineteen patients with more than 1 year of postoperative follow-up at the time of publication were evaluated when comparing preoperative and 12 months or more later. Differences were considered statistically significant at a value of P<0.05.

## RESULTS

Operative characteristics are indicated in Table-2. The mean operative time was 208.3±48 min., while the mean blood loss was 75.8±28.9 mL. Omentum and perirenal fat were used to cover the graft in 21 and 3 cases, respectively, and the decision was made intraoperatively based on omentum accessibility. The length of the hospital stay was 7.3±2.5 days. No intraoperative complications were observed. Postoperatively, seven patients (29.2%) developed complications. Five of the patients experienced urinary tract infections leading to the prescription of additional antibiotics (Grade II). Two patients developed a Grade IIIa complication, which included leakage of the anastomosis site. Anastomosis leaking was diagnosed as a result of profuse drainage. To correct this, temporary percutaneous nephrostomy tube drainage was placed, and control of the leakage severity followed. On the 5th postoperative day, the nephrostomy tube was removed with no signs of leakage on antegrade pyelography.

**Table 2:** Operative and postoperative characteristics.

Parameter		Mean±SD (range)
Operative time, min		208.3±48 (140-300)
Estimated blood loss, mL		75.8±28.9 (50-150)
**Wrap of the buccal graft, n (%)**		
	omental flap	21 (87.5)
	perirenal fat	3 (12.5)
Length of stay, days		7.3±2.5 (3-13)
Follow-up, month		22±12.9 (4-45)
Stricture-free at last visit, n (%)		21/24 (87.5%)
**Complication rate, n (%)**		
	Grade II	5 (20.8)
	Grade IIIa	2 (8.3)

**Figure 4:**
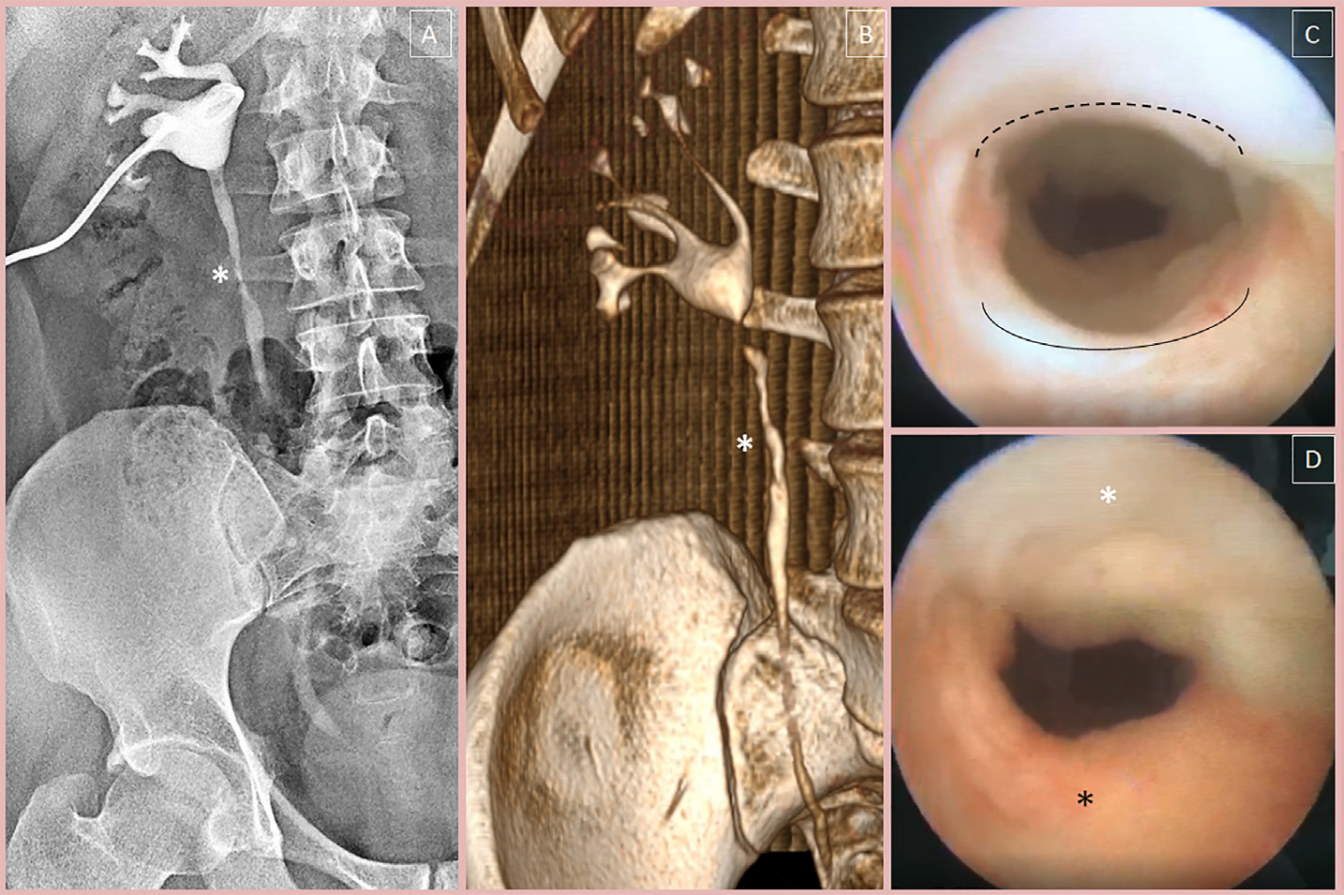
A) Antegrade pyelography indicates patency of ureter after removal of the ureteral stent. B) Three-dimension reconstruction of CTU on 12 months postoperatively. On both pictures, the white asterisk indicates ureteroplasty site. C) Endoscopic view of BMG after 12 months postoperatively. The interrupted and solid line indicates the graft and health ureter in the distal level of BMG ureteroplasty, respectively. D) Endoscopic view of BMG in the stricture site. The white and black asterisk indicates BMG and health ureter, respectively.

Before discharge, all patients underwent excretory pyelography or antegrade pyelography in the presence of a nephrostomy tube. As a follow-up, all patients were advised to see a doctor if even modest complaints appeared, as well as to perform computed tomography-urography (CTU) every six months during the first two years and annually after. Moreover, 12 months after the intervention, all patients underwent ureteroscopy to visualize ureteroplasty (Figure-4). The mean follow-up was 22±12.9 (4-45) months. At the time of writing this study, the stricture-free rate was 87.5% (21 of 24). Notably, two patients with stricture recurrence on the 21st and 31st-month of follow-up had a previous failed uretero-ureteral anastomosis and were managed with balloon dilatation followed by double-J stent indwelling. The decrease in serum creatinine was statistically significant (Table-3). There was a difference between the frequency of grade 0/1 and grade 2/3 hydronephrosis in the pre- and postoperative periods (p<0.001).

**Table 3:** Pre- and postoperative parameters.

Parameter		Before surgery, n=24 (a)	after removal of drains, n=24 (1,5-2 months)	Follow up after 12 months or more, n=19 (b)	P-value (a vs b)
Serum creatinine (μmol/L)		86.6±22.8	82±17.9	80.3±19.5	0.021*
**Pain, n (%)**					
	Yes	18 (75)	3 (12.5)	2 (10.5)	<0.001*
	No	6 (25)	21 (87.5)	17 (89.5)	
**SFU grade of hydronephrosis, n (%)**					
	Grade 0	0	14 (58.3)	13 (68.4)	<0.001*
	Grade 1	0	7 (29.2)	4 (21.1)	
	Grade 2	17 (70.8)	2 (8.3)	2 (10.5)	
	Grade 3	7 (29.2)	1 (4.2)	0	
	Grade 4	0	0	0	

Values are mean±SD.

* Statistically significant difference.

## DISCUSSION

Long proximal ureter strictures remain challenging for surgeons (10, 11). End-to-end anastomosis with a tension-free and watertight state is not always possible in these cases, and in the current literature, such cases are proposed to be cured via ureteral replacement with ileal, downward nephropexy or auto-transplantation (12–14).

Ileal substitution of the ureter is a challenging procedure with a significant rate of complications due to intestinal resection and postoperative metabolic changes. Nephropexy and auto-transplantation of the kidney in a downward fashion are also not easy-to-perform surgical procedures and are often associated with complications, such as pseudoaneurysms and vascular thrombosis, which results in their relatively rare use (15, 16). Given the current modality drawbacks, developing surgical techniques that may facilitate the management of proximal ureteral strictures is recommended.

Buccal mucosa graft is actively used for reconstructive urethral surgery (17). The buccal mucosa is readily available for harvesting, less prone to immune reactions, and tolerates urinary tract pathogens. BMG ureteroplasty also becomes more and more popular, especially when a tension-free anastomosis is difficult to achieve via ureteroureterostomy or for patients with recurrent ureteral strictures who previously underwent failed ureteroplasty associated with peri-ureteral scarring and poor ureteral vascularization (18). According to the recent review, the overall reported success rate was reported to be 66 out of 72 (91.6%), 32 out of 34 (94.1%) and 34 out of 38 (89.5%) open and robotic cases, respectively. The complication rate was reported in 60 cases, being 15 out of 60 (25%) for all complications, with a 5% (3/60) rate for complications graded as Clavien–Dindo score ≥III (19).

Most of the current studies on minimally invasive ureteroplasty in BMG have focused on the robot-assisted procedure (6, 7, 18, 19). Arora et al. reported no data for recurrence during the 6-month follow-up period (20). More recently, Zhao et al. published the results of robot-assisted BMG ureteroplasty in 19 patients recruited from three clinics in the United States. In 74% of these cases, the stricture was localized in the upper third, while the remaining 24% of patients had a middle ureteral stricture. The length was about 4 cm (2-8 cm). The onlay technique cured 79% of patients, and the rest underwent augmented anastomotic ureteroplasty. The total success rate was 90% for an average 26-month follow-up period (7).

Despite the encouraging results of performing BMG ureteroplasty via robotic surgery, it has a downside. On the one hand, robot-assisted surgery is not as popular as desired. The international community recognized that the optimal use of robotic technology requires the development of dedicated training pathways and that outcomes during the learning curve should be scrutinized (21). Data not only on open and robotic but also laparoscopic BMG ureteroplasty should be available in literature. On the other hand, most hospitals throughout the globe have just shifted their armamentarium from open to laparoscopic surgery, and the latter should not be omitted even for challenging procedures. However, it should be noted that performing reconstructive operations of the urinary tract using laparoscopic access requires expert mastery of laparoscopic techniques, including intracorporeal suturing.

To expand the current data on laparoscopic BMG ureteroplasty, we conducted a retrospective study on 24 patients with proximal ureteral stricture who were amenable to laparoscopic surgery. According to our results, even this challenging procedure could be performed via laparoscopy. The operative time, complication rate and intraoperative parameters were optimal. There was no significant difference compared with previously published results of robotic BMG ureteroplasty.

Our study has several limitations that should be noted. First, the retrospective nature may lead to some bias compared to a prospective study. Second, we reported only results from ventral onlay because it is more convenient for laparoscopic surgery. In contrast, previous data on robotic results comprise the mixed data on dorsal and ventral onlay, and some maneuvers accessible for the robotic ureteroplasty are challenging in laparoscopy. Third, we collected data from one hospital, which could also lead to bias associated with the surgeons’ experience in this field. Nevertheless, this study is valuable because it helps to reveal the theoretical feasibility of laparoscopic BMG ureteroplasty. Additional experience and multi-clinical studies are needed to clarify the role of laparoscopic BMG ureteroplasty in ureteral reconstructive surgery.

## CONCLUSION

Laparoscopic ventral onlay ureteroplasty with a buccal mucosa graft could effectively treat patients with proximal ureteral strictures. However, it should be noted that in order to perform this type of ureteroplasty effectively, a careful approach to patient selection is necessary. Also, have experience in various reconstructive surgeries on the upper urinary tract, including ileal ureter substitution, and be prepared to perform them. Since the final decision on the type of reconstructive surgery is made intraoperatively.

## ABBREVIATIONS

BMG =: buccal mucosa graft
LOS =: length of stay
SD =: standard deviation
SFU =: The Society for Fetal Urology
CTU =: computed tomography – urography
BMI =: body mass index
